# Case report of nipple shield trauma associated with breastfeeding an infant with high intra-oral vacuum

**DOI:** 10.1186/s12884-015-0593-1

**Published:** 2015-07-26

**Authors:** Sharon L. Perrella, Ching T. Lai, Donna T. Geddes

**Affiliations:** School of Chemistry and Biochemistry, M310, The University of Western Australia, 35 Stirling Highway, Crawley, WA 6009 Australia

**Keywords:** Intra-oral vacuum, Nipple shield, Nipple pain

## Abstract

**Background:**

Nipple pain is associated with early cessation of breastfeeding and may be caused by high intra-oral vacuum. However identification of high intra-oral vacuum is typically restricted to the research setting. This is the first reported case of an infant with high intra-oral vacuum that was clinically identified through a specific pattern of nipple trauma associated with nipple shield use. Knowledge of clinical signs associated with high intra-oral vacuum may facilitate early recognition of this unusual breastfeeding challenge.

**Case presentation:**

The mother of an exclusively breastfed 3 month old infant had severe bilateral nipple pain with minimal trauma that persisted from birth. The nipples were not misshapen immediately after breastfeeding and adjustments to infant attachment at the breast did not attenuate the pain. Examination of the infant’s oral anatomy was unremarkable with no ankyloglossia present. Microbiological cultures of nipple swabs and breast milk were negative for bacterial and fungal growth, and prescribed antimicrobial treatments did not reduce the nipple pain. Mild blanching and erythema of the nipples were occasionally observed, and were not consistent with nipple vasospasm. Nipple shields were used regularly as they modified the pain, although this resulted in blisters that corresponded with the nipple shield holes. Measurement of infant intra-oral vacuum during breastfeeding confirmed intra-oral vacuum up to 307 % higher than reference values. Breastfeeding gradually became less painful, and after 6 months was completely comfortable.

**Conclusions:**

High intra-oral vacuum is difficult to assess in the clinical setting and is likely an under-reported cause of early weaning that is not well understood. This original case report highlights high intra-oral vacuum as at differential diagnosis to be considered by health professionals when evaluating mothers experiencing strong nipple pain during the initiation of breastfeeding. A clinical screening tool is needed to enable prompt identification of these infants.

## Background

Nipple pain is a significant contributor to early cessation of breastfeeding [1] and so rapid and appropriate management is imperative. Common causes include suboptimal attachment of the infant to the breast, ankyloglossia, infection, dermatitis, and nipple vasospasm [2]. In the research setting, high intra-oral vacuum has been identified as a cause of persistent nipple pain; infants with high vacuum exert baseline and peak vacuums 30 to 60 % greater than that measured in infants with uncomplicated breastfeeding [3, 4]. It is recognized that intra-oral vacuum is integral to successful breastfeeding, with a baseline vacuum of −64 ± 45 mmHg required to sustain attachment and peak vacuum −145 ± 58 mmHg applied during milk removal [3]. However the mechanisms of intra-oral vacuum and the etiology of vacuum anomalies are not well understood. In the absence of a screening tool, clinical identification of high intra-oral vacuum is problematic. We describe the case of a breastfeeding dyad where the mother had an unusual pattern of nipple trauma related to nipple shield use, which led to diagnosis of high intra-oral vacuum in the infant.

## Case presentation

A 35 year old primiparous woman had persistent and severe nipple pain from the onset of breastfeeding. The medical, family and psychosocial history was unremarkable with no breast or nipple trauma, surgery or piercings, and the woman expressed motivation to breastfeed.

The female infant was born at 37.1 weeks gestation after induction for fetal growth restriction. The birth weight, 2600 g, was on the 10^th^ centile and subsequent weights tracked along the 5^th^ centile of the World Health Organization weight-for-age girls percentile chart [5]. The infant remained healthy and developmentally appropriate.

Due to attachment difficulties, hand expressed colostrum was syringe fed to the infant until secretory activation on the fourth postnatal day when a nipple shield was introduced and breastfeeding commenced. Nipple pain was experienced at the first breastfeed, and continued during and randomly between breastfeeds. The pain was described as “pinching, tight and raw pain” and superficial nipple trauma occurred regularly. Mild blanching and erythema of the nipples were infrequently observed and did not match the typical clinical picture of nipple vasospasm. Some breastfeeds were less painful than others, and regular paracetamol (acetaminophen) reduced the pain marginally.

In the second postnatal month severe burning and shooting pains were experienced in the right breast during and after feeds. Nipple swabs were taken for microbiological culture and concurrent prescribed courses of oral flucloxacillin and fluconazole were completed. The breast pain abated within a few days of taking the medications, but the nipple pain continued.

Nipple shields (24 mm, Medela AG, Baar, Switzerland) were used regularly as they modified the pain, although health professionals had repeatedly discouraged this due to concerns about an association between nipple shield use and reduced breastmilk supply. Use of an electric breast pump (Swing, Medela AG, Baar, Switzerland) on the default (average −53 mmHg) or slightly higher vacuum setting felt “tender rather than painful…really low, low level pain…nothing like breastfeeding.”

During the first three postnatal months several consultations were undertaken with an obstetrician, a community child health nurse, international board certified lactation consultants, and family physicians. Bacterial infection and candidiasis were considered likely diagnoses and so nipple swabs were sent for microscopy and culture on three occasions. However no pathogenic growth was detected and neither courses of oral flucloxicillin nor oral fluconazole had an effect on nipple pain. (Details of the drug doses and course durations are not available). Ultrasound examination was conducted to exclude nipple and breast pathology, and no abnormalities were detected. Wearing of breast warmers (Promix HB, Solma, Sweden) in cold weather provided some comfort between feeds.

At 3 months the mother and infant attended the practice of the first author in a final attempt to identify the cause of persistent, severe nipple pain. The infant had been exclusively breastfed from birth and was feeding every 3 to 4 h during the day with an overnight inter-feed interval of 6 h. Examinations of the infant’s mouth, maternal breasts and nipples were unremarkable. Test weighing was performed before and after a clinically assessed breastfeed. Positioning and attachment at the breast appeared satisfactory, and the mother was in obvious pain; pale, sweating and barely able to talk throughout the breastfeed. Immediately post feed the nipple was not compressed and there was mild areolar edema radiating 1 cm from the nipple base. The infant fed from both breasts transferring 136 mL in 10 min.

Feeding with a nipple shield was reported to be less painful although it intensified pain in the nipple tip with a sensation of “…the nipple being drawn out through the end of the shield.” Further questioning revealed the formation of blisters on the nipple tips that correlated with each of the nipple shield holes.

The mother was invited to complete measurement of her 24 h milk production and to attend a research facility for measurement of the infant’s intra-oral vacuum. The 24 h breastmilk production was measured by test-weighing the infant before and after each feeding from each breast on an electronic scale (BabyWeigh™, Medela Inc, McHenry IL, USA, resolution 2 g, accuracy ± 0.034 %) for a period of 24 h plus one breastfeeding. A corrected 24 h production for each breast then was determined with measurements of breastfeed amounts and milk production expressed in grams that is considered to be nearly equivalent to mL. Normal milk production was confirmed (Table 1) [6].

**Table 1:** Breastfeeding and 24 h breast milk production profile for an exclusively breastfeeding dyad with high intra-oral vacuum in the infant

	Case	Population average (range)^a^
Number of breastfeeds	12	11 (6 – 18)
Average (range) volume (mL)	62 (60 – 172)	76 (30 – 135)
Average duration (min)	8	16 (5 – 37)
24 h breastmilk intake (mL)	740	
Expressed milk (mL)	50	
Total 24 h milk production	790	788 (478 – 1357)

^a^Kent et al. [6]

**Fig. 1:**
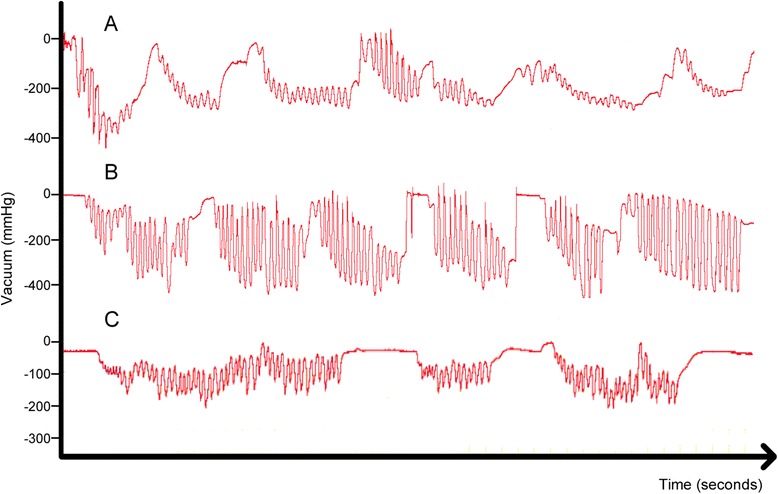
Intra-oral vacuum traces for infant with high vacuum during (**a**) direct breastfeeding and (**b**) breastfeeding with a nipple shield, and (**c**) infant with expected range of intra-oral vacuum

**Fig. 2:**
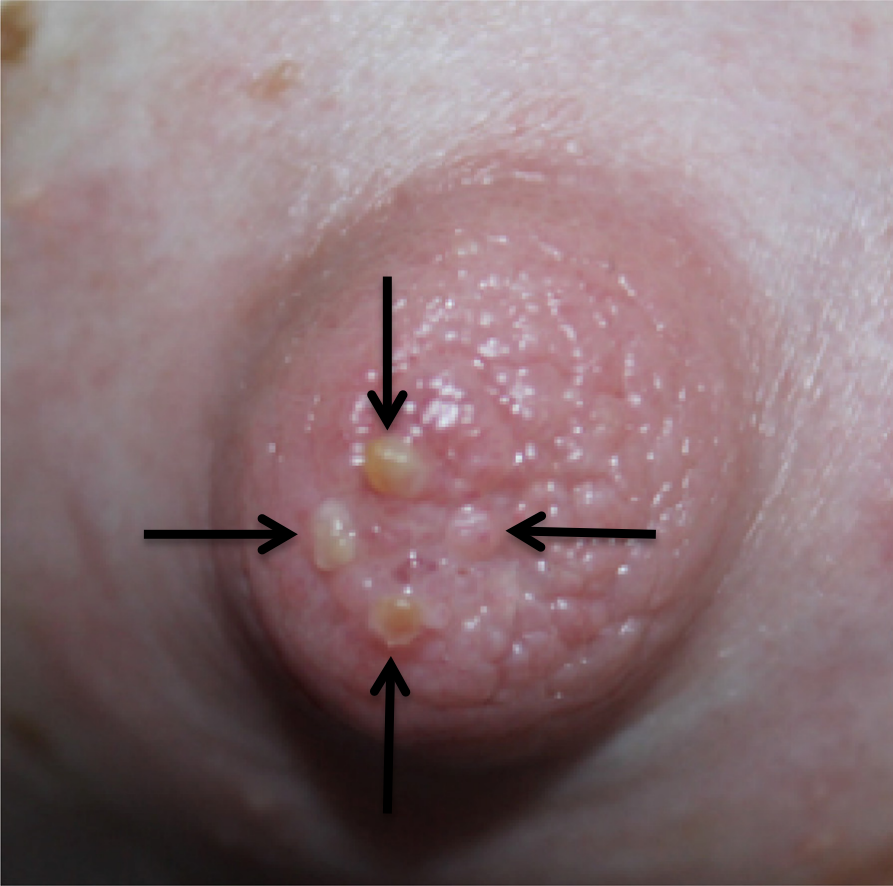
Blisters resulting from nipple shield use while breastfeeding an infant with high intra-oral vacuum

Intra-oral vacuum was measured during breastfeeding using a small silicone tube (Supplemental Nursing System, Medela AG, Baar, Switzerland) filled with sterile water with one end positioned alongside the mother’s nipple and the other end attached *via* a silicone tube (650 mm, 4 mm) and a 3-way tap to a pressure transducer (SP854, Memscap, Bernin, France) with disposable clip-on dome (MLA844, AD Instruments, Castle Hill, Australia). High intra-oral vacuum was measured both during active sucking and when pausing on the breast, both with and without use of the 24 mm Medela nipple shield, (Table 2, Fig. 1). During direct breastfeeding average peak, baseline and pausing vacuums were 43, 107 and 307 % higher than reference values [4]. Nipple shield use appeared to normalize baseline and pausing vacuums, however peak vacuums were 207 % higher, and blisters were observed on the nipple tips immediately after feeding (Fig. 2). Sub-mental ultrasound observation of tongue movements during breastfeeding demonstrated compression of the nipple base when the tongue depressed during milk flow.

**Table 2:** Intra-oral vacuum measurements during direct breastfeeding (breast) and breastfeeding with a nipple shield. Values are reported as mean ± standard deviation (range)

	Reference range^a^	Breast	Nipple shield
Sucking vacuum mmHg			
Peak	−163 ± 62	−233 ± 152 (−128, −360)	−338 ± 32 (−278, −368)
Baseline	−56 ± 31	−151 ± 62 (0, −83)	−41 ± 37 (−3, −74)
Mean		−183 ± 56	−169 ± 54
Duration (s)	12.2 ± 4.8	12.5 ± 12.4	9.2 ± 5.8
Pausing vacuum mmHg			
Mean	−46 ± 30	−141 ± 73 (−21, −233)	−40 ± 70 (0, −157)
Duration (s)	4.4 ± 2.6	1.7 ± 0.6 (0.87, 1.8)	1.2 ± 0.44 (0.9, 2.2)

^a^McClellan et al. [4]

The following management strategies were offered to limit or alter exposure to high-intra-oral vacuum during breastfeeding.

1. Remove infant from the breast when non-nutritive sucking begins towards the end of a breastfeed.
2. Replace some breastfeeds with bottle feeds of expressed breastmilk.
3. Trial alternative breastfeeding positions (such as lying down) to vary the location of nipple base pressure.
4. Trial a larger nipple shield to reduce the intra-oral space and therefore reduce vacuum as per Boyle’s law.

Follow up at 4 months revealed that the mother replaced some breastfeeds with feeding of expressed milk to rest her nipples, and when convenient she breastfed lying down as it varied the location of pain at the nipple base. The mother reported that she felt better able to cope once she was provided with a definitive reason for the pain and some options for managing it. The nipple pain had reduced with use of a 28 mm Mamivac conical nipple shield (KaWeKo, Ditzingen, Germany). However while more comfortable than a 24 mm nipple shield, the nipple skin was still being drawn through the holes of the shield. The family was not available for a follow up measurement of the infant’s intra-oral vacuum when using the larger nipple shield. They gradually weaned from the nipple shield during the fifth month and by 6 months breastfeeding had become completely comfortable and enjoyable. The mother later reported that she achieved her goal of breastfeeding for 12 months.

### Discussion

While the distinct pattern of nipple trauma suggested an intra-oral vacuum anomaly, the ability to measure intra-oral vacuum during breastfeeding was key in identifying the cause of this mother’s extreme and continuing nipple pain. Early diagnosis and management of breastfeeding problems is important to prevent early weaning. However in the absence of a clinical screening tool for intra-oral vacuum, a differential diagnosis of high intra-oral vacuum may not be considered. It is likely that a proportion of these cases wean due to unexplained painful breastfeeding. Current recommendations for management are restricted to reducing the frequency and/or duration of breastfeeds to limit exposure to high intra-oral vacuum.

While evidence for the effect of high intra-oral vacuum on nipple pain is emerging [4], its etiology is not well understood. As peak intra-oral vacuum during breastfeeding coincides with downward movement of the posterior tongue and soft palate [3] it is possible that anomalous tongue or palatal movements contribute. The growing infant’s oral cavity enlarges vertically so that the tongue, which fills the oral cavity of a newborn, occupies a lower proportion of the oral space in older infants [7]. It is not known whether the changes in oral anatomy impact intra-oral vacuum over time and may explain gradual resolution of symptoms of high intra-oral vacuum in the reported case. Further studies that include simultaneous intra-oral vacuum measurements and ultrasound imaging in infants of different ages will further our knowledge of this phenomenon.

Differing vacuum patterns were observed between direct breastfeeding and nipple shield use, with the latter associated with higher peak and normalized baseline and pausing vacuums. While it is not known whether nipple shield use impacts intra-oral vacuum for all infants, altered baseline vacuum may explain in part how nipple shield use facilitates sustained attachment in preterm infants [8].

## Conclusions

In the absence of a clinical measure of infant intra-oral vacuum during breastfeeding, this anomaly is not readily identified and is likely under-diagnosed. High intra-oral vacuum can contribute to early cessation of breastfeeding and so may be considered as a differential diagnosis for breastfeeding women with nipple pain that has persisted from birth. A clinical tool is needed to enable screening for high intra-oral vacuum in cases of persistent bilateral nipple pain.

## Consent

Written informed consent was obtained from the patient for publication of this case report and the accompanying images.
